# High hopes, hard realities: cannabidiol shows no acute effects in youth with alcohol use disorder

**DOI:** 10.1038/s41386-025-02160-w

**Published:** 2025-07-03

**Authors:** L. Cinnamon Bidwell

**Affiliations:** University of Colorado Boulder, Boulder, CO USA

**Keywords:** Drug development, Translational research

Cannabidiol (CBD) is often marketed as a cure-all, from anxiety to addiction, and preclinical studies suggest it may reduce alcohol intake, craving, and relapse [1]. But until recently, no human studies had tested CBD in adolescents and emerging adults with alcohol use disorder (AUD), a population uniquely vulnerable to long-term harm and poorly served by existing treatments. In the first controlled study of its kind, Kirkland and colleagues rigorously evaluated the acute effects of a single 600 mg dose of CBD in youth with AUD [2]. The results were clear: no significant changes in neural, psychological, physiological, or behavioral outcomes, despite robust task engagement and precise methodological control. While null, these findings provide a critical foundation for future translational work, and a cautionary tale about relying too heavily on adult or animal data to guide interventions for youth.

Kirkland et al. conducted a randomized, double-blind, placebo-controlled crossover study in 33 non-treatment-seeking youth (ages 17–22) with DSM-5 AUD [2]. Each participant received 600 mg of oral CBD or placebo (Epidiolex vs. matched sesame oil solution), separated by a minimum 18-day washout. Assessments were timed to coincide with peak expected plasma levels (2–3 hours post-dose), and participants consumed a high-fat snack beforehand to maximize CBD bioavailability.

A multi-modal battery was used to assess CBD’s effects across relevant domains of AUD pathophysiology:

*Proton magnetic resonance spectroscopy (1H-MRS)*. Measured glutamate+glutamine (Glx) and GABA+ levels in the dorsal anterior cingulate cortex (dACC), implicated in addiction-related neurocircuitry.

*Functional MRI (fMRI) alcohol cue-reactivity task*. Quantified BOLD response to alcohol versus non-alcohol beverage cues.

*Psychophysiological olfactory cue task*. Captured craving and autonomic responses (heart rate variability and skin conductance) to alcohol-related and control odors.

*Ecological momentary assessment (EMA)*. Monitored drinking behavior for 7 days post-dosing.

Key Findings from Kirkland et al. include the following:

*No CBD-related effects across outcomes*. CBD did not significantly alter neurometabolite levels, neural cue reactivity, physiological responses, self-reported craving, or short-term drinking behavior compared to placebo. These findings held across both whole-brain and targeted region-of-interest analyses.

*Cue reactivity was robust, but unmodulated by CBD*. Alcohol-related cues elicited craving in self-report measures, but did not alter physiological arousal. CBD did not attenuate these responses, suggesting limited acute impact on cue-induced processes in this age group.

*Neurometabolite levels reflected AUD severity, but not medication effects*. Exploratory analyses linked higher binge drinking and AUD symptom counts with lower Glx and GABA+ levels in the dACC, consistent with literature identifying neurometabolic disruption in AUD. However, CBD had no measurable effect on these markers.

*CBD was well tolerated*. No adverse events were attributed to CBD, reinforcing its safety in this demographic and supporting feasibility for future longitudinal trials.

**Why this null trial still matters**. While the findings were null, they are highly informative. Negative results in early-phase pharmacotherapy trials are essential to refining hypotheses and guiding future research. However, null results can prompt broader concerns about the translational gap in AUD medication development, often referred to as the ‘valley of death’ in the field [3].

*First clinical trial of CBD in youth with AUD*. Although CBD is widely discussed as a treatment for substance use, its efficacy and safety in adolescents and young adults have been virtually untested. Kirkland et al. provide the first human laboratory data in this population, addressing a critical gap between preclinical promise and clinical practice [2].

*Null results with high translational value*. Despite the lack of efficacy, this study offers valuable lessons. The absence of effects may reflect developmental differences in pharmacodynamics, insufficient dosing, or limitations in task sensitivity. Prior studies in adults with AUD using higher or repeated doses have reported changes in neural cue reactivity and craving [4, 5], suggesting that acute administration may be too limited to observe effects in youth.

*Methodological rigor sets a new standard*. The study’s crossover design, time-locked assessments, and multi-modal battery offer a model for early-phase pharmacotherapy testing in youth. The use of ethically appropriate cue tasks (olfactory instead of taste), non-treatment-seeking participants, and comprehensive adverse event monitoring enhance both safety and translational relevance.

*Clarifies future directions*. Future work should explore chronic or dose-escalation of CBD in its designs. Preclinical studies have shown that repeated administration of CBD may be necessary to influence relapse-related behaviors [1], supporting the need for longer-term human clinical trials. Also, incorporation of plasma CBD measurement will be vital to assess exposure variability to better link dose-response relationships in outcomes. In addition, development and use of developmentally tailored cue paradigms (e.g., taste or social-contextual cues) will be crucial to move this line of research forward. The limited salience of standardized stimuli, particularly in fMRI and olfactory tasks, may have muted the potential CBD effects in this study.

## Conclusion

CBD continues to hold potential as a pharmacotherapy for substance use disorders, but this study serves as a necessary checkpoint in the drug development pipeline. Kirkland et al. offer a carefully controlled, methodologically sophisticated investigation that demonstrates the feasibility, but not the efficacy, of acute CBD in youth with AUD [2]. As the field moves forward, these results emphasize the need for age-specific trials, careful attention to dosing and cue salience, and a commitment to publishing negative findings. In doing so, they help close the translational gap and bring the field one step closer to effective, evidence-based treatments for adolescent AUD.
